# Intravitreal treatment in patients with exudative age-related macular degeneration and visual acuity ≤ 0.05

**DOI:** 10.1186/s12886-015-0123-y

**Published:** 2015-10-21

**Authors:** Raphael Koch, Matthias Schmidt, Sabine Gebauer, Holger Busse, Constantin E. Uhlig

**Affiliations:** Institute of Biostatistics and Clinical Research, University of Muenster, Schmeddingstraße 56, 48149 Münster, Germany; Department of Ophthalmology, University of Muenster Medical Center, Albert Schweitzer Campus 1, Building D15, 48149 Münster, Germany

## Abstract

**Background:**

To investigate intravitreal treatment efficiencies in patients suffering from exudative ARMD with a BCVA ≤ 0.05.

**Methods:**

Retrospective analysis: Analysis parameters were lesion type, BCVA at baseline and at follow-up, the intravitreal drug used, and its application frequency. Patients were divided into: 1) following injections of bevacizumab, triamcinolone, their combination, or ranibizumab regardless of their lesion subtype, 2) or by lesion subtype. Statistical tests were performed using Wilcoxon signed-rank tests, Kruskal-Wallis tests and multivariable logistic regressions.

**Results:**

Seventy four eyes of 74 patients were analyzed. Follow-up was at 12.0 to 15.7 weeks. Median difference of BCVA (logMAR) between baseline and follow-up was 0.000 (−0.030, 0.175) in classic (*p* = 0.105), 0.000 (−1.15, 0.20) in occult (*p* = 0.005), −0.200 (−1.20, 0.60) in cases with subretinal fluid (*p* = 0.207), 0.000 (-0.60, 0.30) in pigment epithelial detachment (*p* = 0.813), and 0.050 (−0.40, 0.70) in Junius Kuhnt maculopathy (p = 0.344). BCVA increased ≥ 0.2 logMAR in 4 (24 %) classic, 9 (47 %) occult, 6 (33 %) pigment epithelial detachment, 6 (55 %) subretinal fluid, in 29 (39 %) eyes regardless of the lesion type, and reached a BCVA ≥ 0.05 in 7 (9 %) of those with a baseline BCVA <0.05.

**Conclusions:**

Results indicate that in patients with ARMD and a BCVA lower 0.05, intravitreal treatment may improve visual acuity, most probably in cases with occult lesions.

## Background

Age-related macular degeneration (ARMD) has become one of the most threatening ophthalmologic diseases in patients older than 50 years from developed countries such as the United States or central Europe [1]. Standard treatment of exudative ARMD is repeat intravitreal injection of anti-vascular endothelial growth factor (VEGF) antibody, ranibizumab, aflibercept, or pegabtanib, which are legally accepted but expensive. Because of increased age of the general population, insurance companies are increasingly less likely to pay for treatment for all patients [2]. Therefore, a few years ago, insurance companies requested a minimal best corrected visual acuity of ≥ 0.05 to financially cover ARMD treatment with photodynamic therapy in Germany. Actually, the recommendation of professional ophthalmological associations in Germany (Deutsche Ophthalmologische Gesellschaft, Retinologische Gesellschaft, Berufsverband der Deutschen Augenärzte) to treat exudative ARMD are not exclusively reduced to the BCVA, but a BCVA of ≥ 0.05 is still a landmark, and evidence based scientific investigations reporting about such patients with low vision are missing. In other countries the costs of these treatments is an issue, too [3, 4]. Specifically, in these cases, intravitreal use of bevacizumab, which costs much less, about one twentyfifth of ranibizumab, is a reasonable but off-label option. Several prospective controlled studies analyzing BCVA development following intravitreal injection of bevacizumab or ranibizumab have already been published [5, 7–9]. However, unfortunately these studies do not analyze treatment results in patients with BCVA lower than 0.05, probably because such inclusion criterias, a minimal BCVA of 0.05 or 0.0625, have been comparatively adapted since the beginning of photodynamic treatment with verteporfin in 2003 in all major studies, e.g. TAP, VIP, FOCUS, ANCHOR, MARINA, PIER, or IVAN [10–16].

The primary aim of our analysis was to investigate whether intravitreal treatment in exudative ARMD and BCVA ≤ 0.05 had improved visual acuity in our patients.

## Methods

This is a retrospective analysis of patients suffering from exudative ARMD with BCVA ≤ 0.05 at baseline, who received intravitreal injections of bevacizumab, ranibizumab, triamcinolone, or in combination at our University hospital. Following detailed information about scientifically established pros and cons, including side effects, e.g. possible secondary glaucoma or cataract using triamcinolone, treatment regimen were choosen depending on the patients’ economic possibilities and final options. Treatment regimen did not follow a strict rule. The patients were fully informed about the experimental character of the treatments and had given written informed consent.

Analyses adhered to the Declaration of Helsinki, and, due to its retrospective character, an approval of the study protocol was not necessary by the Ethics committee of the Medical Association of Westphalia-Lippe and of the medical faculty of the Westphalian Wilhelms-University, Muenster, Germany.

Retrospective analysis parameters were age and gender of the patients, the type of exudative ARMD and best corrected visual acuity (BCVA) tested with Snellen letters at a distance of 5 meters and for VAs lower than 0.05 at 1 meter. BCVA had always been documented at baseline and at follow-up, as well as the intravitreal drug given, its application frequency, and dose. Inclusion criteria were age ≥ 50 years and diagnosis of exudative ARMD with a BCVA ≤ 0.05. Counting fingers was set at 0.013, and hand motion at 0.005 [15, 16]. A gain of ≥ 1 Snellen line was defined as an improvement in BCVA, and a loss of ≤ 1 Snellen line as a decrease in BCVA. Additionally, we evaluated the change in VA of 0.2 difference in logMAR which generally indicates two line difference in ETDRS chart.

Exclusion criteria were excentric vision, a history of vitrectomy, any treatment with bevacizumab, pegabtanib, or ranibizumab within the last six months, any intravitreal application of triamcinolone within the last twelve months, or a follow-up of less than 12 weeks.

The following groups were analyzed for BCVA:

After injection of bevacizumab, triamcinolone, ranibizumab or in combination,

1. regardless of the subtype of choroidal neovascular membrane (CNV),
2. according to the subtype of CNV (classic or occult),
3. patients without possible CNV classification presenting subretinal fluids,
4. patients without possible CNV classification presenting also pigment epithelial detachment,
5. patients without CNV classification presenting macular scars and hemorrhages, defined as Junius-Kuhnt maculopathy.

Statistical analyses were performed using IBM SPSS® Statistics 22 for Windows (IBM Corporation, Somers, NY, USA). Inferential statistics were intended to be exploratory instead of confirmatory, and were interpreted accordingly, meaning that p-values were only used to generate new hypotheses. Thus, neither a global significance level nor local levels were determined, and no adjustment for multiplicity was made. P-values represent a metric measure of evidence against the respective null hypothesis, and were considered as statistically noticeable if *p* ≤ 0.05.

Standard univariate statistical analyses were used to describe demographical and clinical parameters. Categorical variables are shown as absolute and relative frequencies. Continuous variables, which were assumed to be normally distributed, are presented as mean ± standard deviation. Non-normally distributed metric variables are reported as median (minimum; maximum). Comparisons of BCVA changes between baseline and follow-up were conducted using exact nonparametric Wilcoxon signed-rank tests. Changes were calculated by subtracting baseline BCVA from follow-up-BCVA. Comparisons of BCVA between groups of different lesion subtypes were conducted using Kruskal-Wallis test. Additionally, BCVA (decimal acuity) was translated to logMAR and analyzed using the same methods.

A multivariable logistic regression was calculated to detect possible influences on the binary target parameter improvement of BCVA in decimal acuity (BCVA ≥ 0.05 at follow-up). Independent parameters were age in years, gender, and lesion type (classic lesion, occult lesion, subretinal fluids, pigment epithelial detachment, Junius-Kuhnt maculopathy).

## Results

Because of the inclusion and exclusion criteria, the follow-up of 74 eyes from 74 patients with ARMD could be analyzed. The mean age of these patients was 81.9 years (median 83.5, range 58–96 years), with 47 eyes from female and 27 from male patients. Follow-up was at 12.0 to 15.7 weeks following intravitreal treatments. CNV could be classified in 36 cases (Table 1), 17 of which were classic, 19 occult. In 38 eyes CNV could not be definitely classified anymore and edemas, i.e. subretinal fluids were observed (*n* = 11), pigment epithelial detachments (*n* = 9), macular hemorrhages (*n* = 4), or typical Junius-Kuhnt maculopathy (*n* = 8).

**Table 1:** Descriptive measures concerning age and gender of patients, and development of BCVA in different ARMD subtypes following treatment

	All types of exsudatlve ARMD		Classic lesion		Occult lesion		Subfoveal fluid		Pigment epithelial detachment		Junlus-Kuhnt maculopathy	
Number of eyes (n)	74		17		19		11		9		8	
Gender (n): male/female	27/47		10/7		6/13		3/8		2/7		3/5	
Patient age in years:												
Mean	81.9		79.5		80.6		85.9		83.7		81.9	
Standard deviation	7.6		8.9		8.1		5.5		4.2		5.9	
Median	83.5		83.0		83.0		85.0		84.0		84.0	
Minimum	58.0		58.0		59.0		77.0		76.0		70.0	
Maximum	96.0		91.0		90.0		96.0		91.0		87.0	
BCVA at baseline:	decimal	logMAR	decimal	logMAR	decimal	logMAR	decimal	logMAR	decimal	logMAR	decimal	logMAR
Mean	0.042	1.42	0.039	1.46	0.047	1.34	0.040	1.44	0.042	1.41	0.035	1.54
Standard deviation	0.130	0.22	0.015	0.26	0.009	0.14	0.014	0.21	0.014	0.21	0.017	0.34
Median	0.050	1.30	0.050	1.30	0.050	1.30	0.050	1.30	0.050	1.30	0.040	1.40
Minimum	0.005	1.30	0.005	1.30	0.013	1.30	0.013	1.30	0.013	1.30	0.005	1.30
Maximum	0.050	2.30	0.050	2.30	0.050	1.90	0.050	1.90	0.050	1.90	0.050	2.30
BCVA at follow-up:												
Mean	0.082	1.27	0.065	1.33	0.138	1.04	0.077	1.26	0.061	1.36	0.029	1.69
Standard deviation	0.097	0.40	0.057	0.38	0.158	0.38	0.080	0.38	0.058	0.36	0.020	0.44
Median	0.050	1.30	0.050	1.30	0.050	1.30	0.050	1.30	0.050	1.30	0.030	1.55
Minimum	0.005	0.15	0.005	0.70	0.025	0.15	0.013	0.50	0.013	0.70	0.005	1.30
Maximum	0.700	2.30	0.200	2.30	0.700	1.60	0.300	1.90	0.200	1.90	0.050	2.30
Difference of BCVA between baseline and posttherapy:												
Mean	0.040	−0.15	0.026	−0.13	0.091	−0.30	0.038	−0.17	0.019	−0.06	−0.006	0.15
Standard deviation	0.096	0.36	0.057	0.31	0.157	0.40	0.086	0.28	0.054	0.37	0.012	0.19
Median	0.000	0.00	0.000	0.00	0.000	0.00	0.015	−0.20	0.000	0.00	−0.005	0.05
Minimum	−0.040	−1.20	−0.030	−0.90	−0.015	−1.15	−0.037	−1.20	−0.025	−0.60	−0.020	−0.40
Maximum	0.650	0.70	0.175	0.40	0.650	0.20	0.028	0.60	0.150	0.30	0.010	0.70
Cases (n/%) of BCVA < 0.05 at baseline and > 0.05 at final visit	7/9 %		2/12 %		1/5 %		2/18 %		0/0 %		1/13 %	
Cases (n/%) with a decrease of BCVA of > 0.2 logMAR	29/23 %		4/24 %		9/47 %		6/55 %		3/33 %		1/13 %	

### Analysis of BCVA following injections of bevacizumab, triamcinolone, in combination, or ranibizumab regardless of the subtype of CNV, i.e. including classic, occult and mixed forms

Intravitreal injections with either bevacizumab or triamcinolone were performed in 56 patients’ eyes. When bevacizumab was used (*n* = 24), BCVA increased in eleven patients (with eleven patients having an increase of ≥ 0.2 logMAR), remained stable in seven patients, and decreased in six patients.

When triamcinolone was used (*n* = 32), BCVA increased in thirteen, remained stable in fifteen, and decreased in four patients (with eight patients having an increase of ≥ 0.2 logMAR).

Regardless of the subtype of CNV (Table 1), the median difference in BCVA between posttherapy and baseline was 0.000 (−0.040; 0.650, *p* < 0.001) in decimal acuity and 0.00 (−1.20; 0.70, *p* = 0.001) in logMAR . In seven patients, BCVA in Snellen letters exceeded 0.05 at final visit, though it had been less than 0.05 at baseline.

## Analysis of BCVA according to the subtype of CNV

**Fig. 1:**
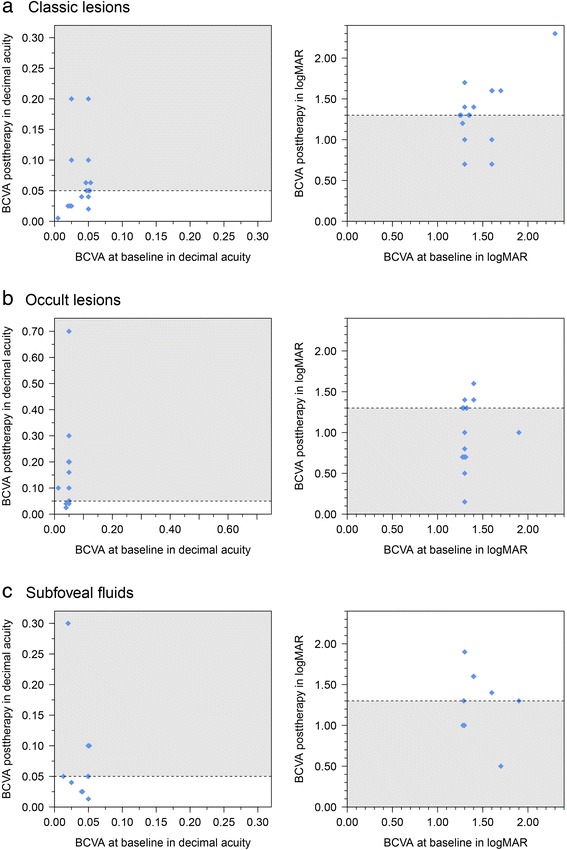
Development of BCVA in patients with exsudative ARMD and (**a**) classic subtype, (**b**) occult subtype, and (**c**) in cases with subfoveal fluids without subtype differentiation. Grey area defines cases with visual acuity that have increased and exceeded 0.05 decimal acuity (Snellen letters, left side) or gone below 1.3 logMAR (right side) during follow-up. Due to equal values (ties), a random offset was added to these values to enhance visibility
- Occult CNV (*n* = 19):
  In patients with occult CNV and intravitreal injection of bevacizumab, triamcinolone, or in combination, or ranibizumab, BCVA (Snellen letters) improved in nine cases (all of which exceeded a BCVA (Snellen letters) of 0.05, and nine increased ≥ 0.2 logMAR, (47.4 %)), remained stable in eight cases, and decreased in two cases (Fig. 1b).

- Classic CNV (*n* = 17):
  If patients received bevacizumab, triamcinolone, a combination, or ranibizumab, BCVA increased in seven, remained stable in eight, and decreased in two cases. The median differences between posttherapy and baseline in BCVA were 0.000 (−0.030; 0.175, *p* = 0.074) in Snellen letters and 0.00 (−0.90; 0.40, *p* = 0.105) in logMAR. Two patients exceeded 0.05 BCVA (decimal acuity) posttherapy, and four patients (24 %) had an increase of ≥ 0.2 logMAR. If triamcinolone was injected once (*n* = 11), BCVA improved in five and remained stable in four cases (Fig. 1a). Two patients had an increase of ≥ 0.2 logMAR.

Following injection of bevacizumab (*n* = 4), BCVA remained stable in two patients, and increased in two patients. Following injection of triamcinolone (*n* = 12), BCVA increased in five cases, all of whom had an increase of ≥ 0.2 logMAR , remained stable in six and decreased in one case. The median differences of BCVA in decimal acuity 0.000 (−0.015; 0.650) and logMAR 0.00 (−1.15; 0.20) were statistically significant (*p* = 0.005, *p* = 0.005). One patient who had a visual acuity of < 0.05 at baseline exceeded 0.05 at final visit (Table 1).

### Analysis of BCVA in patients with late stage ARMD and subfoveal fluids

Because of the late stage of ARMD, CNV subtype could not be definitely classified any more.

**Fig. 2:**
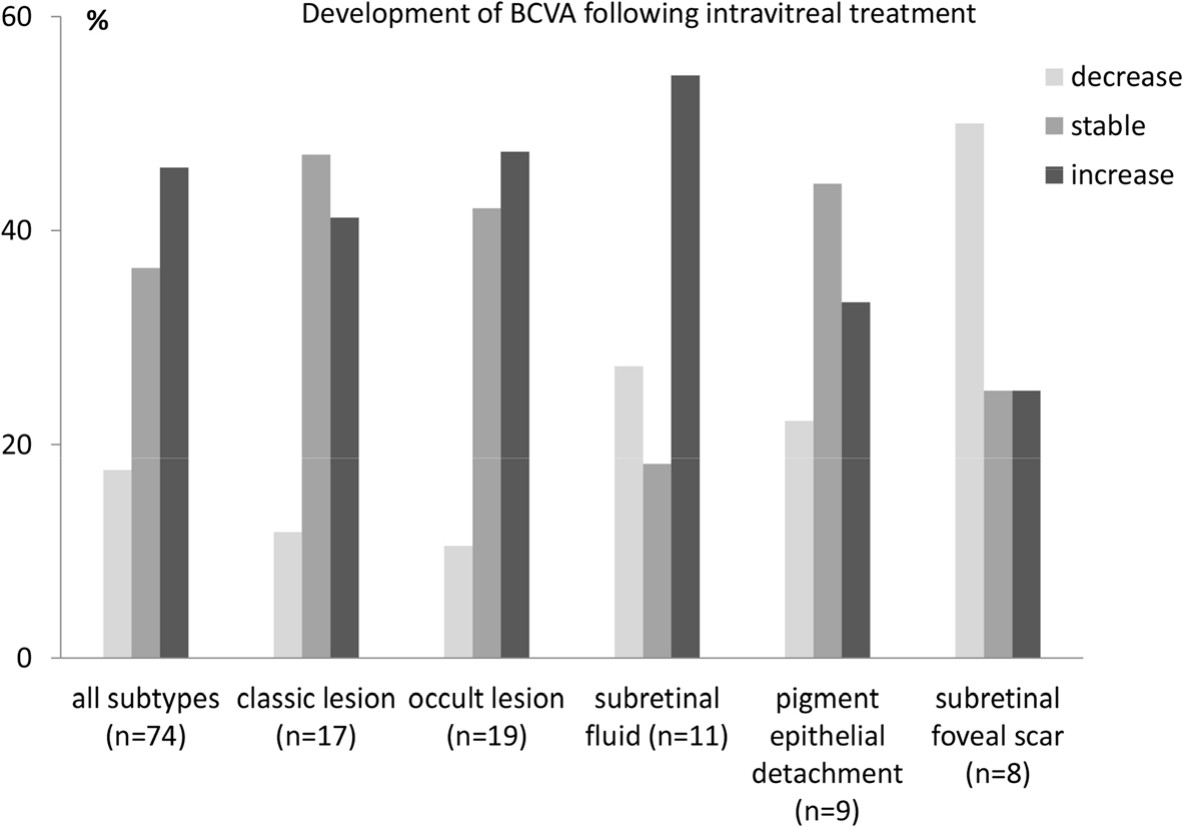
Development of BCVA in different ARMD subtypes during follow-up

BCVA decreased in three eyes, remained stable in two, and improved in six cases, all of these with an improvement ≥ 0.2 logMAR (55 %) following intravitreal injection (single or multiple injections of bevacizumab, triamcinolone, combination, or ranibizumab). In two eyes, BCVA reached or exceeded 0.05 though it had been lower than 0.05 at baseline (Fig. 1c). The median differences between final visit and baseline of BCVA in Snellen letters 0.015 (-0.037; 0.280) and logMAR −0.20 (−1.20; 0.60) were not statistically noticeable in patients with late stage ARMD and subretinal fluids (*p* = 0.098; *p* = 0.207). Nevertheless, increases in BCVA during follow-up were observed most often in these patients (Fig. 2).

### Analysis of BCVA in patients without definite CNV classification presenting pigment epithelial detachment

**Fig. 3:**
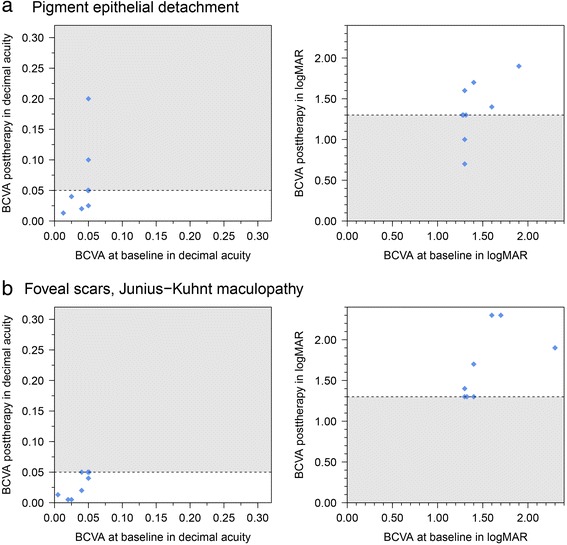
Development of BCVA in patients with exudative ARMD and (**a**) pigment epithelial detachment, and (**b**) Junius-Kuhnt maculopathy. Grey area defines cases with visual acuity that have increased and exceeded 0.05 decimal acuity (Snellen letters, left side) or gone below 1.3 logMAR (right side) during follow-up. Due to equal values (ties), a random offset was added to these values to enhance visibility

Similar to patients with subretinal fluids, it was not possible any more to define CNV subtype. BCVA increased in three eyes. In no case BCVA exceeded 0.05, if it had been less at baseline. BCVA remained stable in four cases and decreased in two cases (Fig. 3a). The median differences between posttherapy and baseline in decimal acuity (0.00; −0.025; 0.150, Table 1) and in logMAR (0.00; −0.600; 0.30) were not noticeable (*p* = 0.625; *p* = 0.813).

### Analysis of BCVA in patients with Junius Kuhnt maculopathie including subretinal foveal scars

Due to subretinal hemorrhages, and scars, it was not possible to define CNV subtype any more. BCVA increased in two patients (one with an increase of ≥ 0.2 logMAR), remained stable in two patients and decreased in four patients (Fig. 2). The median differences in BCVA Snellen letters and logMAR between posttherapy and baseline were −0.005 (−0.020; 0.010; *p* = 0.188) and 0.05 (−0.40; 0.70; *p* = 0.344) respectively (Table 1, Fig. 3b).

Descriptive statistics showed a BCVA increase of 41 % in patients with classic lesions, 47 % (occult), 33 % (pigment epithelial detachment), 55 % (subretinal fluids), and in 46 % regardless of the lesion type. In 29 patients (39 %) an increase of ≥ 0.2 logMAR was observed, and in 7 (27 %) patients with baseline BCVA < 0.05, BCVA reached or exceeded 0.05.

The difference in BCVA development between different lesion types (classic, occult, late stage ARMD with subretinal fluids, pigment epithelial detachment, or foveal scar/Junius-Kuhnt maculopathy) was statistically not noticeable (decimal acuity *p* = 0.169; logMAR *p* = 0.190). Multivariable analysis using logistic regression showed no noticeable influence of the parameters gender, age and lesion type on the improvement of BCVA.

## Discussion

In practice, many patients consult their ophthalmologist when their BCVA has already decreased to less than 0.05. At this time it is difficult to recommend a definite procedure because it may not be clear whether such therapy is efficient in these advanced cases, due to the very little information that is actually known [19].

Nevertheless, the Eyetech Study Group reported from their phase 1A study of 5 of 6 patients with a baseline BCVA of <0.05 (mean 0.025) which then increased to a mean of 0.036 on day 84 following treatment with pegabtanib [20]. In their phase II study, they also presented results of 3 patients with a baseline BCVA <0.05 (mean 0.035) which all increased following treatment to a mean of 0.1 [21].

But in both studies, case numbers were too small and lesion types have not been classified to allow specific characterization.

Sørensen and Kemp, instead, analyzed retrospectively 33 patients suffering from age-related macular degeneration which presented an increase of mean BCVA from logMAR 1.3 (range 1.1–2) to 1.0 (range 0.3–2) following treatment with intravitreal ranibizumab [19]. Unfortunately, the authors did not specify the lesion subtypes.

The Comparisons of Age-Related Macular Degeneration Treatments Trials (CATT) Research Group has published a two-year study, comparing the intravitreal efficacy of ranibizumab with bevacizumab in exudative ARMD with equivalent effects on BCVA. However, the study also focused only on patients with a minimal BCVA of 0.0625 [5], and Biswas and coworkers who observed equal effects regarding the efficiencies of bevacizumab and ranibizumab in the treatment of neovascular ARMD did not include low vison patients neither [6].

In a retrospective analysis of the treatment of 48 eyes of 47 patients, which presented a baseline BCVA between 20/150 till hand movements (logMAR 1.34 ± 0.25), Ehrlich and coworker described a BCVA increase to 20/50 till countingfingers (logMAR 1.2 ± 0.42) with a BCVA improved by ≥3 lines in 25 % following intravitreal application of bevacizumab [22].

In our retrospective investigation, BCVA exceeded 0.05 in 9 % of patients with baseline BCVA <0.05 irrespective of the subtype of exudative ARMD. And we observed a BCVA increase in nearly one third of all patients irrespective of the subtype of exudative ARMD. BCVA increased most often in occult lesions and in those cases, where subtypes could not be classified any more, but presented subretinal fluids. We cannot prove but presume that these maculopathies have been originated from former occult lesions, as well, since well demarcated lesions were not seen.

Though Jonas et al. and Tao & Jonas did not observe a correlation between the lesion subtype and the development of BCVA in ARMD, our investigation supports the hypothesis that in patients with exudative ARMD with BCVA < 0.05, retinas with occult CNV benefit more than those with classic lesions [23, 24]. Patients with Junius-Kuhnt maculopathy presented most often a decrease in BCVA, and mean BCVA was comparably lowest during follow-up which is reasonable, because these maculopathies often present foveally located scars. Due to small sample size, treatment efficiency of eyes with mixed CNV, or macular hemorrhage were not analyzed.

The usefulness of our study is limited by its retrospective nature, a relatively small number of patients, the heterogeneity of intravitreally applied drugs and frequencies, the partial difficulty in diagnosing a lesion subtype, and the fact that many patients were not able to pay and stopped repetitive treatment, even when intravitral medication improved BCVA. As we did not have any control group, it was not possible to exclude a placebo-like effect.

## Conclusions

Similar to Galbinur et al. who retrospectively analyzed patients with wet ARMD and a BCVA of 0.1 or worse [25], our results suggest that for patients with ARMD and a BCVA < 0.05, intravitreal treatment may improve visual acuity, specifically in cases with occult lesions. We therefore suggest to specify such outcomes in late stages of ARMD also with the use of an EDTRS or a Radner chart [26, 27], including investigation of contrast or glare sensitivity and color vision, or adaptation to darkness [17] which could emphasize such treatment benefits. As the use of intravitreal steroids have been reported to be only helpful in the short-term follow-up [28], such investigations should primarily focus on Anti-VEGF agents to characterize the most useful intravitreal drug and the baseline threshold of BCVA for which treatment is still significantly helpful.
